# The cancer-associated secretory phenotype: a new frontier in targeted therapeutics

**DOI:** 10.1172/JCI182652

**Published:** 2024-09-03

**Authors:** Xiaochao Tan, Guan-Yu Xiao, Priyam Banerjee, Shike Wang, Jonathan M. Kurie

**Affiliations:** 1 Department of Medicine, Tulane University School of Medicine, New Orleans, Louisiana, USA.; 2 Department of Toxicology and Cancer Biology, University of Kentucky College of Medicine, Markey Cancer Center, Lexington, Kentucky, USA.; 3 Bio-Imaging Resource Center, The Rockefeller University, New York, New York, USA.; 4 Department of Thoracic/Head and Neck Medical Oncology, The University of Texas MD Anderson Cancer Center, Houston, Texas, USA.

Following the acquisition of oncogenic mutations, cells undergo senescence and acquire heightened secretory states termed senescence-associated secretory phenotypes (SASPs). Proteins identified in SASPs include a broad range of inflammatory and immune-modulatory cytokines and chemokines, growth factors, and cell surface molecules. In seminal work from the laboratory of Judith Campisi, genotoxic stress was shown to induce senescence partly via SASPs (1). Oncogene expression also induces senescence, and proteins secreted in response to genotoxic stress or oncogenic RAS expression demonstrate a high degree of overlap (1). In work from the laboratory of Dafna Bar-Sagi, mutant RAS activates secretion in established cancer cells that gain tumorigenic activity following ectopic mutant RAS expression (2). Therefore, mutant RAS activates secretion in both senescent and nonsenescent cells. More recently, we identified other oncogenic signals that activate secretory programs in cancer cells, including p53 loss, chromosome 1q and 3q amplicons, and epithelial-mesenchymal transition (3–6). For the purposes of this discussion, oncogene-activated secretory programs in nonsenescent cancer cells will collectively be referred to herein as cancer-associated secretory phenotypes (CASPs).

## Distinguishing CASP from SASP

SASPs and CASPs mediate their extracellular actions through partially overlapping sets of secreted effectors (1), but their upstream regulators appear to be distinct. Genotoxic stress activates SASPs through the transcription factor NF-κB, which drives the expression of interleukin-6 and -8, indicating that heightened expression of secreted effectors sustains SASPs. Not surprisingly, SASPs are also regulated by classical protein secretory pathway components, including PKD1, ARF1, and PI4KIIIβ (7). In contrast, somatic mutations activate CASPs by initiating cargo-specific secretory vesicle biogenesis in the Golgi and accelerating anterograde trafficking of secretory vesicles from Golgi to plasma membrane (3, 4, 8). Embedded within the CASP regulatory network are Golgi-resident protein complexes that govern cargo sorting, Golgi membrane bending, and vesicle scission (3, 4, 8, 9). Cargo-sorting proteins recognize and load specific cargos into secretory vesicles (10). In line with these observations, CASPs govern the secretion of specific cargos (3–5).

## Secretory blockade as a therapeutic strategy

**Figure 1:**
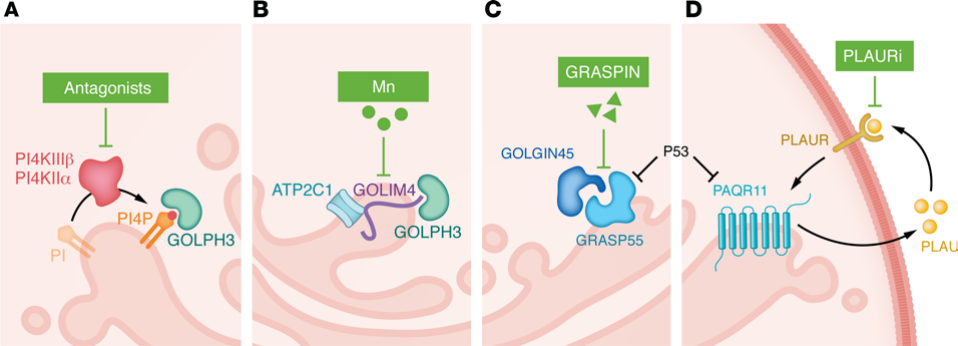
Blockade of secretory vesicle biogenesis provides a therapeutic strategy for targeting cancer. (**A**) Phosphatidylinositol-4-phosphate (PI4P) is the Golgi membrane insertion site for Golgi phosphoprotein 3 (GOLPH3), a pivotal regulator of Golgi membrane dynamics and vesicle biogenesis. Golgi PI4P is generated by two Golgi-localized phosphatidylinositol 4-kinases (PI4Ks): PI4KIIIβ and PI4KIIα. PI4K antagonists block PI4P synthesis and impair secretion. (**B**) Golgi scaffold GOLIM4 recruits effectors of vesicle biogenesis (GOLPH3) and cargo sorting (ATP2C1) to activate secretion. GOLIM4 is degraded by manganese (Mn), resulting in diminished secretion. (**C**) P53 loss increased the expression levels of the Golgi scaffold GRASP55. GOLGIN45 is a client of GRASP55 and generates a protein complex that promotes vesicle biogenesis. GRASPIN disrupts the interaction between GRASP55 and GOLGIN45. (**D**) The Golgi scaffold PAQR11 is induced upon the loss of P53. PAQR11 promotes the secretion of PLAU, which functions in an autocrine manner by binding to the PLAU receptor (PLAUR). Activated PLAUR, in turn, promotes the expression of PAQR11. PLAUR inhibitor (PLAURi) prevents the binding of secreted PLAU to PLAUR, effectively terminating an autocrine signaling pathway that drives secretion.

Based on evidence from preclinical models (11), antibodies and decoy receptors designed to neutralize individual secreted effectors in the extracellular space were tested in patients with cancer (11). These approaches demonstrated limited efficacy (11). While multiple factors could have contributed to these disappointing outcomes, functional redundancy within the cancer secretome may have negated any antitumor activity of the neutralization approaches. Therefore, strategies to block the entire cancer secretome, rather than the actions of single peptides in the extracellular space, warrant consideration. In line with evidence that the cancer secretome contains a broad range of effectors that maintain cancer cell survival and drive immunosuppression and fibrosis in the tumor microenvironment (3, 12), secretory blockade induces apoptosis in tumor cells, restores antitumor immunity, and reverses acquired resistance to immune checkpoint inhibitor therapies in preclinical models (12). Consequently, strategies have been developed based on an understanding of how oncogenic mutations interface with the conventional secretory pathway (Figure 1).

Secretory vesicle biogenesis in the Golgi requires phosphatidylinositol-4-phosphate (PI4P), the Golgi membrane insertion site for Golgi phosphoprotein 3 (GOLPH3), and other proteins that drive vesicle extraction from Golgi membranes (9). Golgi-resident PI4P is generated by two enzymes, PI4KIIIβ and PI4KIIα (13). High levels of these enzymes initiate secretory vesicle biogenesis (3, 5, 14). Selective antagonists of PI4KIIIβ or PI4KIIα induce secretory blockade and tumor regression (3, 5). Sensitivity to these agents is tightly linked to a PI4KIIIβ-encoding chromosome 1q amplicon and a PI4KIIα-upregulating transcriptional program activated by epithelial-mesenchymal transition (Figure 1A) (3, 5).

Golgi-resident scaffolds coordinate client proteins dedicated to a common task (15). The scaffolds Golgi integral membrane protein 4 (GOLIM4) and Golgi reassembly and stacking protein 55 kDa (GRASP55) recruit protein complexes that coordinate cargo loading, Golgi membrane curvature, and vesicle scission to activate CASPs (6, 8). Pharmacologic strategies to degrade GOLIM4 or inhibit GRASP55/client interactions induce secretory blockade and tumor regression (6, 8). Sensitivity to these agents is tightly linked to a GOLIM4-encoding chromosome 3q amplicon (Figure 1B) and a GRASP55-upregulating transcriptional program activated by p53 loss (Figure 1C) (6, 8).

p53 loss leads to high expression of the Golgi scaffold PAQR11 and enhances the secretion of protease urokinase plasminogen activator (PLAU), which activates autocrine signals that accelerate secretory vesicle biogenesis in the Golgi, completing a prosecretory feed-forward loop in p53-deficient cancer cells (4). Pharmacologic or genetic approaches to interrupt the PLAU-dependent autocrine loop block secretion and inhibit tumor growth and metastasis (4). PLAU secretion and sensitivity to PLAU antagonists are tightly linked to p53 deficiency (Figure 1D) (4).

## Candidate secretory antagonists

PI4K antagonists that are under development as antiviral agents inhibit secretion and exert selective antitumor activity against chromosome 1q–amplified malignancies (3). Originally developed as a male contraceptive (16), a small molecule that inhibits GRASP55/client protein interactions inhibits secretion and exerts antitumor activity in p53-deficient tumor models (8). Systemic manganese (Mn) delivery to mice bearing chromosome 3q–amplified malignancies degrades intratumoral GOLIM4, inhibits secretion, and induces tumor regression (6). In a phase I study, intranasal Mn delivery was well tolerated in patients with advanced cancer (17). Given that chromosome 1q and 3q amplicons are largely mutually exclusive from known actionable oncogenic mutations (3, 6), novel agents are needed for chromosome 1q– and 3q–amplified malignancies. It’s important to note that both cancer and healthy cells utilize the same Golgi secretory pathway, suggesting that blocking it could affect noncancerous cells as well. However, cancer cells demonstrate an increased reliance on factors from heightened secretion, driven by the amplification or upregulation of Golgi genes (4–6, 8, 18) or somatic driver mutations (4, 8). This heightened dependency makes cancer cells more sensitive to inhibition of secretion compared with healthy cells, presenting a potential therapeutic window.

## Summary

Oncogenic mutations activate secretory programs that drive tumor progression and represent therapeutic targets. The clinical relevance of CASPs is underscored by their prevalence, their linkages to specific genetic and epigenetic contexts, the proven efficacy of secretory blockade in preclinical models, and the availability of assays to identify vulnerable patient populations.

## Version 1 09/03/2024

Electronic publication
